# Study of hydrothermal processes in ice-layers subgrade under constant temperature and dynamic loading

**DOI:** 10.1038/s41598-024-54089-7

**Published:** 2024-02-17

**Authors:** Jinbang Zhai, Ze Zhang, Linzhen Yang, Kunchao Zhou

**Affiliations:** 1 School of Transportation/Ministry of Education Observation and Research Station of Permafrost Geo-Environment System in Northeast China/Permafrost Institute, Northeast Forestry University, Harbin, 150040 People’s Republic of China; 2 School of Civil Engineering/Institute of Cold Regions Science and Engineering/Collaborative Innovation Centre for Permafrost Environment and Road Construction and Maintenance in Northeast China, Northeast Forestry University, Harbin, 150040 People’s Republic of China; 3 Heilongjiang Provincial Investment Group Co., Ltd., Harbin, 150090 People’s Republic of China; 4 Heilongjiang Transportation Investment Group Co., Ltd., Harbin, 150001 People’s Republic of China

**Keywords:** Hydrothermal, Ice-layers subgrade, Constant temperature, Dynamic loading, Engineering, Civil engineering

## Abstract

The presence of ice-layers in the subgrade soils makes the hydrothermal state of road subgrade built in island permafrost regions more susceptible to external environmental influences. In order to deepen the study of the ice-layers subgrade, a hydrothermal study of subgrade under constant temperature and dynamic loading was carried out. It was found that dynamic loading can change the temperature, moisture and pore water pressure distribution. Under dynamic loading, the hydrothermal and pore water pressure state of the soil in the upper part of the ice layer changed significantly at the beginning of the test. The application of dynamic loads alters the spatial distribution of pore water pressure in the soil, resulting in pressure differences between different areas, which affects the migration of moisture and ultimately leads to the formation of areas with higher moisture content in the area below the load. However, the reduction in soil temperature will weaken the effect of the load, therefore, the temperature of the soil should be controlled for frozen subgrade with ice-layers to prevent the accumulation of moisture in the soil.

## Introduction

Damage to subgrade and buildings caused by temperature changes is a common engineering problem in cold regions^1–3^. Freeze–thaw damage of road subgrade is not only related to engineering geological conditions, but is also strongly influenced by the external environment^4–6^. Road subgrades built in islanded permafrost zones are affected by temperature, but also by traffic loads on the road surface. The repeated action of vehicle loads exacerbates the damage to the subgrade pavement^7^. Under the combined effect of temperature and vehicle loads, uneven deformation of the subgrade pavement occurs, leading to settlement of the subgrade, tilting of the pavement and the generation of longitudinal and transverse cracks^8,9^.

More research has been carried out on the hydrothermal effects of temperature changes on permafrost subgrade. Studies have shown that during the freezing process, moisture migrates towards the freezing front, resulting in a redistribution of moisture in the soil^10,11^. Moisture migration in permafrost is related to the soil water potential gradient and depends mainly on factors such as soil properties, the rate of freezing and the rate of swelling^12^. Studies of load on permafrost have focused on the effect of load on the mechanical properties of permafrost. It was found that there is a linear positive correlation between the dynamic stress of damage in permafrost and the frequency of loading, and that the dynamic strain of permafrost is significantly influenced by frequency^13^. In addition, the dynamic elastic modulus of permafrost is related to the dynamic load frequency^14^. The dynamic strength of permafrost decreases non-linearly with increasing number of load vibrations. Vibrating loads lead to a reduction in strength, but the frequency of vibration does not affect the strength values^15^.

It is clear from the above analysis that there is little research into the hydrothermal of permafrost under load. In order to further deepen the hydrothermal study of ice-layers island permafrost subgrade, a hydrothermal study of permafrost subgrade with ice-layers under constant temperature and dynamic loads was carried out.

## Materials and methods

### Test materials

**Figure 1:**
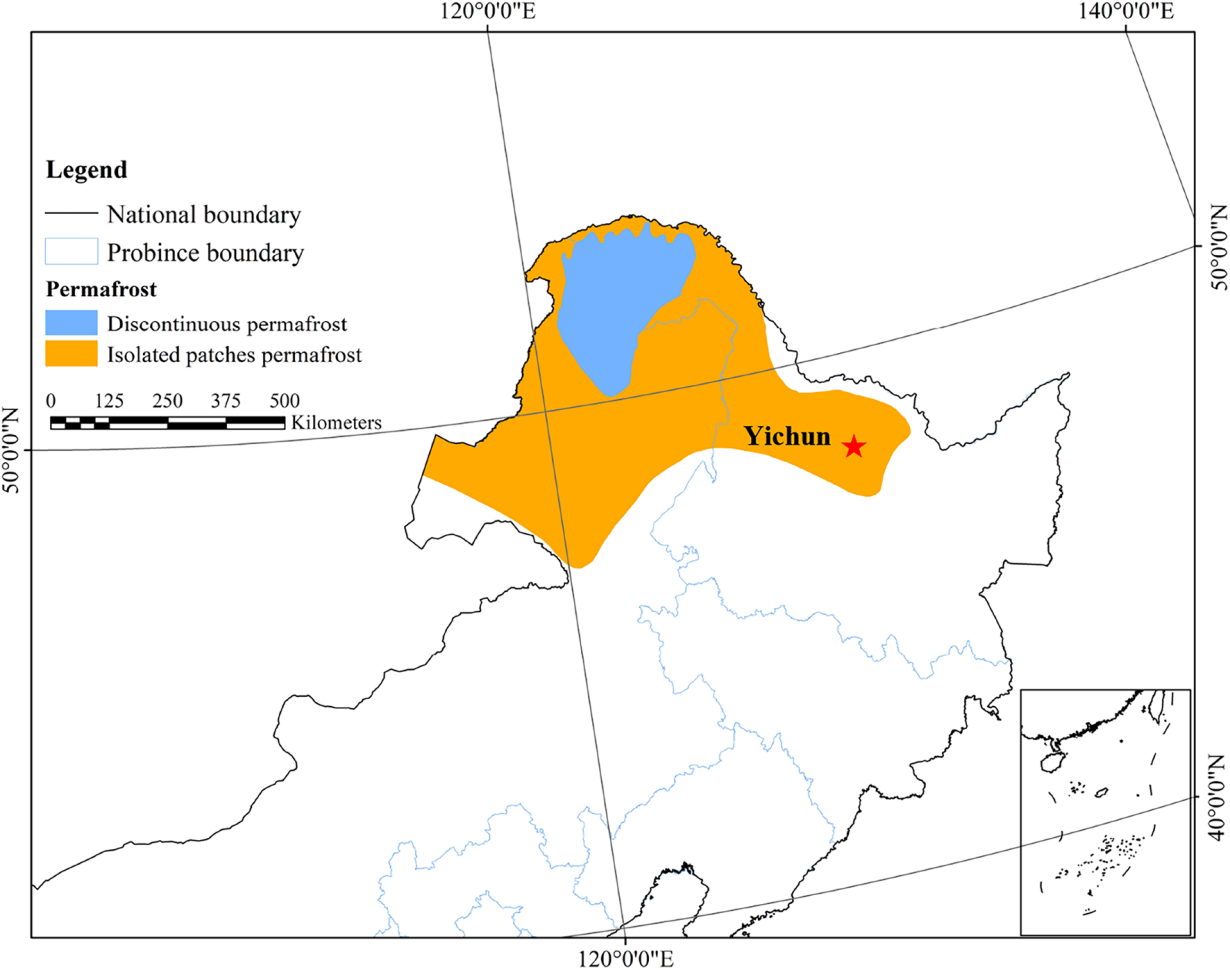
Distribution of permafrost in north-eastern China (Permafrost distribution data is provided by National Cryosphere Desert Data Center. (http://www.ncdc.ac.cn); boundary shapefiles (Drawing number: GS (2019) 1822) is provided by https://www.mnr.gov.cn/).

**Figure 2:**
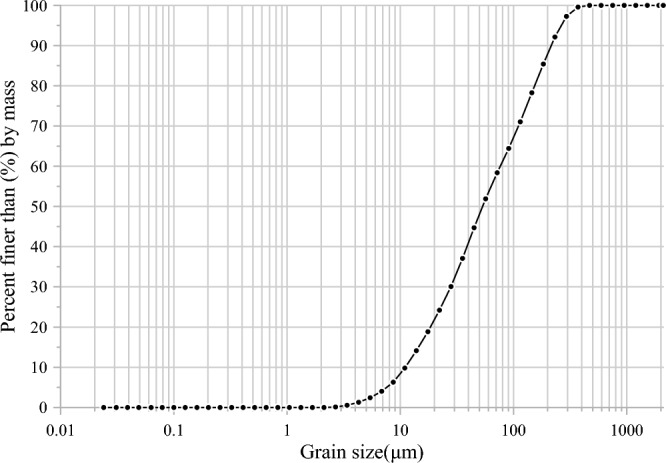
Grain-size distribution of soil.

The test soil samples were taken from the vicinity of the Qiannen Highway in Yichun (Heilongjiang Province, China) and are shown in Fig. 1. The soil sample is located in an area of island permafrost. The initial moisture content of the soil sample was 11.52% and the liquid and plastic limits were 36.50% and 21.77% respectively. The grain size distribution of the soil samples is shown in Fig. 2.

### Test methods

**Figure 3:**
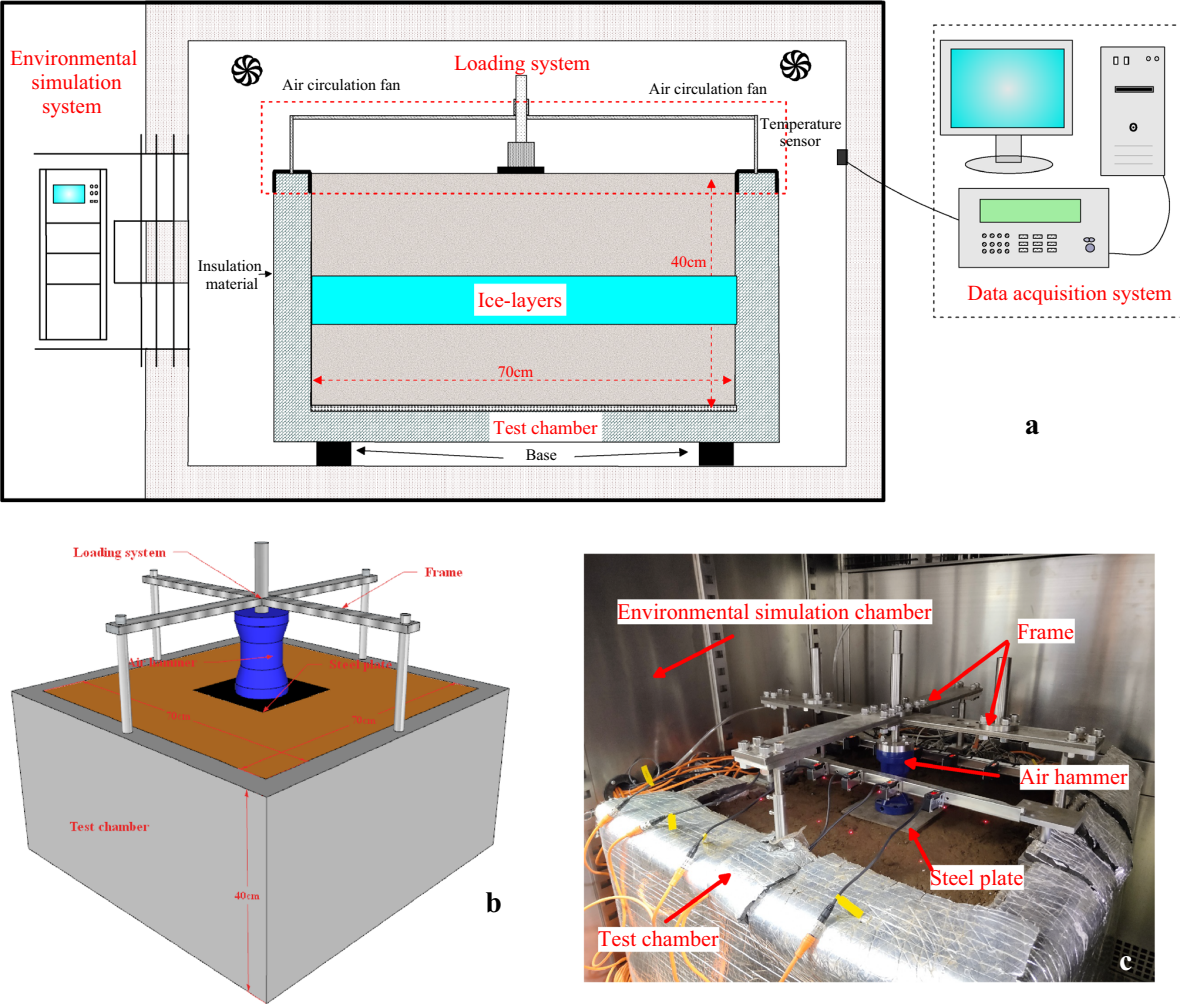
Schematic diagram of the test. (**a**) Schematic diagram of the test structure; (**b**) schematic diagram of the loading system and test box; (**c**) pictures of the test site.

The test consists mainly of an environmental simulation system, a loading system, a test chamber and a data acquisition system, as shown in Fig. 3a. The environmental simulation system has a temperature control range of -40 °C to 150 °C and a temperature control accuracy of 0.1 °C. The loading system consists of an air pump, a control cabinet, a frame and an air hammer, as in Fig. 3b. The process of applying the dynamic load is as follows: the air pump provides the power, which is connected to the control cabinet via a pipe, and the control cabinet is connected to the air hammer via a pipe. The control cabinet allows the setting of the loading time, the loading interval and the loading period. The loading time can be set from 0.01 to 99.99, the loading interval from 1 to 9999 and the loading period from 1 to 9999. The loading time, loading interval and loading period are set according to requirements. The internal dimensions of the test chamber are 70 cm × 70 cm × 40 cm (length × width × height) with a 5 cm thick insulation layer, as in Fig. 3b. The data acquisition system consists of various sensors (Temperature sensor, range: − 30 to  + 30 °C, accuracy: ± 0.05 °C; Moisture sensor, range: 0–100%, accuracy: ± 3%; Pore water pressure sensor, range: − 100 to 200 kPa, accuracy: ± 0.1% F.S (Full range)) connected to the computer via the data acquisition instrument.

**Figure 4:**
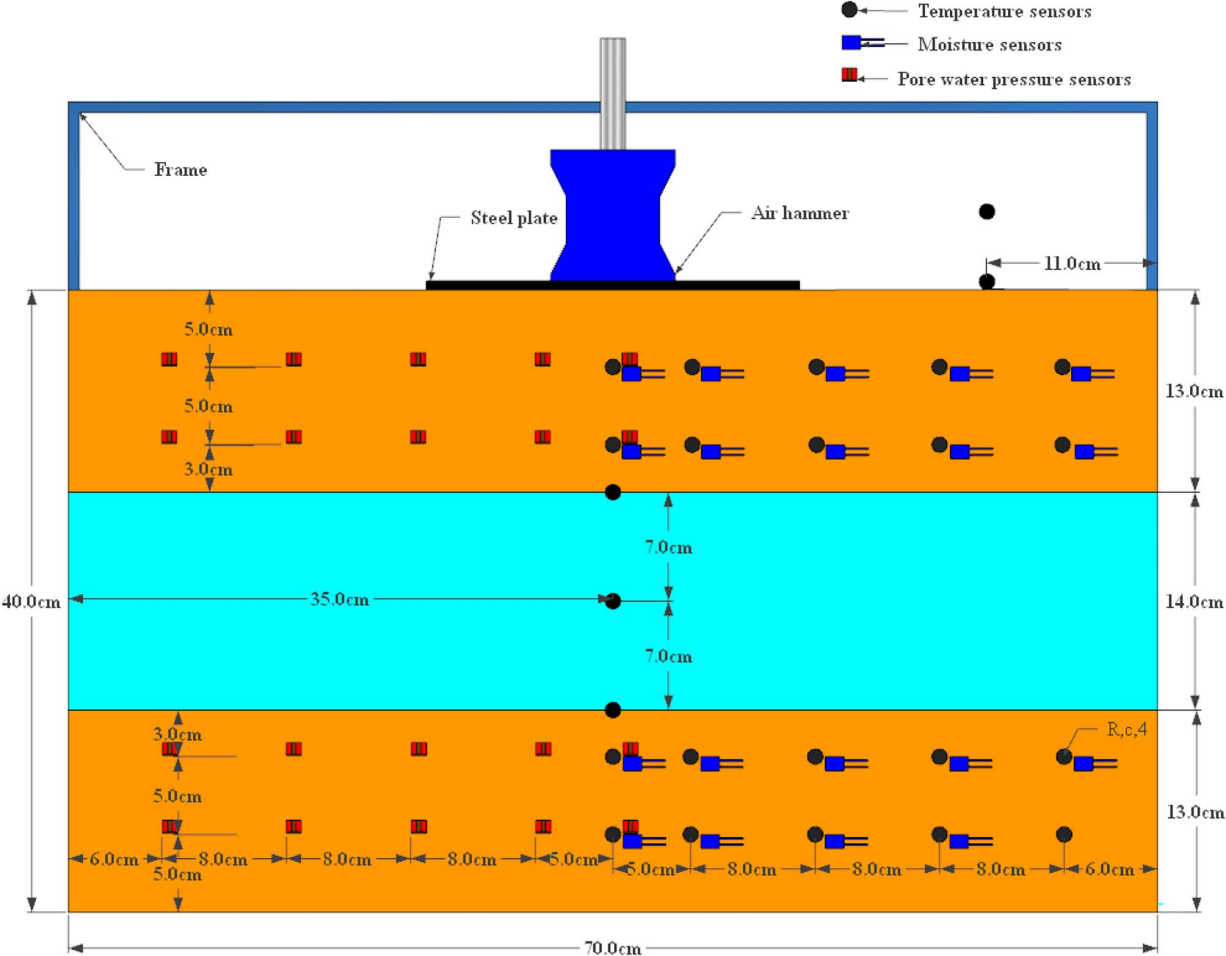
Sensor layout diagram.

The dimensions of the test ice-layer 70 cm in length and width, with the upper soil height, the middle ice layer height and the lower soil height being 13 cm, 14 cm and 13 cm respectively. The soil samples were prepared to a moisture content of 21.77% for use. The prepared soil sample is compacted in layers according to a dry density of 1.6 g/cm^3^. When the lower soil sample has been filled, the lower layer is cooled. The pre-made ice layer is then placed on top of the subsoil sample. The upper soil sample is then filled in. Once the upper soil sample has been filled, the entire soil sample is cooled. Pictures of the test site, as shown in Fig. 3c. The arrangement of the sensors was carried out during the filling of the soil. The sensors were arranged symmetrically, with the pore water pressure sensor on the left side and the temperature and moisture sensors on the right side, and the location of each sensor is shown in Fig. 4.

The temperature control was carried out according to the annual average temperature of − 3.978 °C in Yichun. The load time is the default value of 0.5 s. The load interval is 1 s. The data was collected at an interval of 1 min. As the soil temperature in the island permafrost zone was below 0 °C, the soil was first cooled down and the test started when the overall soil temperature reached below 0 °C. The test was stopped when the lower soil temperature reached − 3.72 °C. Note that at 137.62 h, the air hammer was damaged and replaced, resulting in an increase in ambient temperature. To bring down the ambient temperature, it was adjusted down by 1 °C and continued for 5 h. The test was conducted for a total of 209 h and the data were analyzed at 0 h, 48 h, 96 h, 144 h, 192 h and 209 h.

## Results

### Thermal state evolution during tests

**Figure 5:**
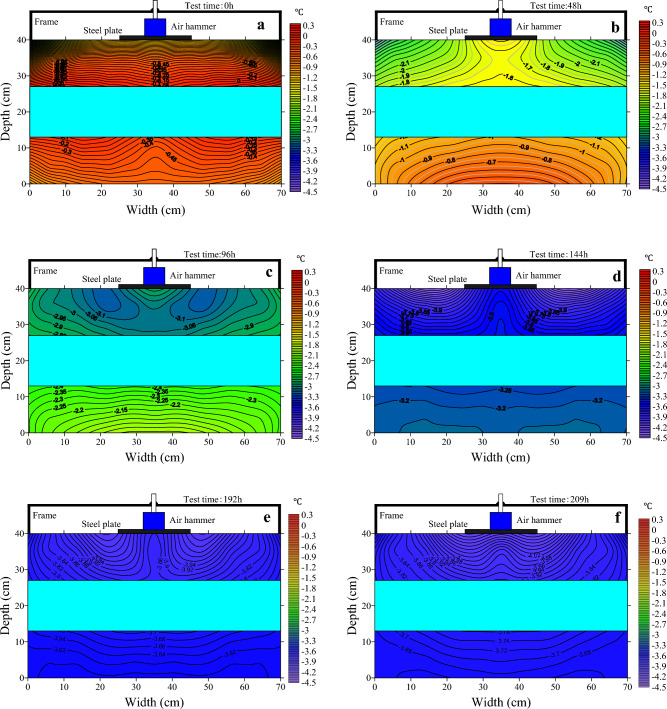
Evolution of the thermal regime over 209 h. (**a**) Temperature field plot at 0 h; (**b**) temperature field plot at 48 h; (**c**) temperature field plot at 96 h; (**d**) temperature field plot at 144 h; (**e**) temperature field plot at 192 h; (**f**) temperature field plot at 209 h.

The reduction in ambient temperature leads to the dissipation of heat from the soil, which in turn leads to a drop in its own temperature. Dynamic loads lead to changes in the soil temperature field through their effect on the basic properties of the soil. In order to analyze the evolution of the temperature field under constant temperature and dynamic loads, the temperature data is analyzed. The evolution of the temperature field at different moments is shown in Fig. 5.

As can be seen from Fig. 5a, the temperature of the soil sample was not uniform, due to the cooling of the specimen prior to the start of the test. After 48 h, as in Fig. 5b, the temperature fields in both the upper and lower soil layers changed compared to 0 h. The temperature field of the upper soil layer under dynamic loading changed differently from that under no loading. The soil temperature is high in the areas with load stress with a value of − 1.5 °C and low in the areas without load stress with a minimum value of − 3.1 °C, a temperature difference of 1.6 °C. On the other hand, the lower soil temperature field presents a different phenomenon to the upper soil. In the lower soil layer, the temperature field in the area below the load does not differ from the rest of the area. The lower temperature in the area close to the test chamber wall is due to the heat transfer from the chamber wall.

The temperature field of the upper soil layer changed further when the test was carried out up to 96 h. From the Fig. 5c, at this time, the temperature value of the soil below the load is − 2.9 °C and the temperature value of the soil on both sides of the load is − 3.15 °C. The cold was transferred from the areas on both side of the load to the area below the load. The overall decrease in temperature in the lower soil layer did not change the spatial distribution significantly. As the test progresses, as in Fig. 5c–f, the cold continues to be transferred from the areas on either side of the load and the area of the upper soil layer where the temperature is high below the load becomes progressively smaller. The overall temperature of the lower soil layer gradually decreases and tends to be the same.

### Moisture evolution during tests

**Figure 6:**
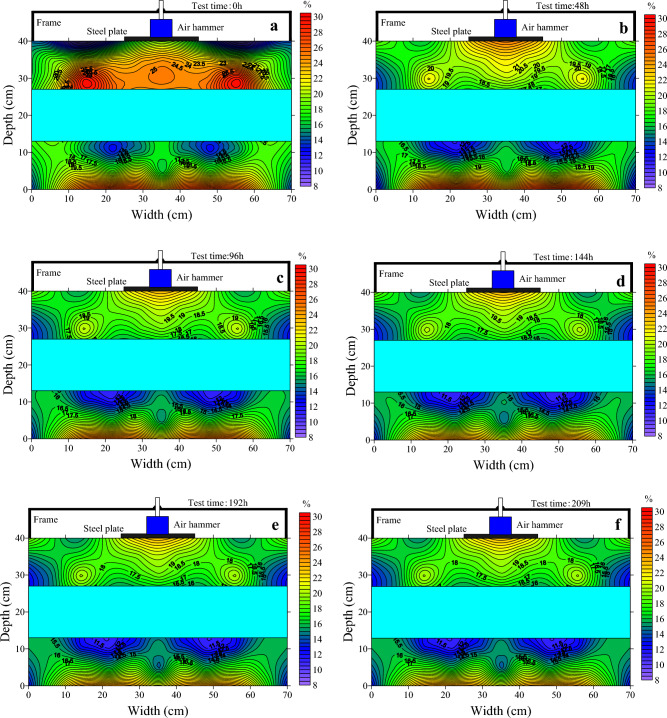
Evolution of moisture over 209 h. (**a**) Moisture field plot at 0 h; (**b**) moisture field plot at 48 h; (**c**) moisture field plot at 96 h; (**d**) moisture field plot at 144 h; (**e**) moisture field plot at 192 h; (**f**) moisture field plot at 209 h.

The decrease in temperature causes the migration of moisture in the soil from the unfrozen zone to the frozen zone, resulting in a redistribution of moisture in the soil. Studies have shown that the spatial distribution of the moisture field within the soil is closely related to the stress field generated by the static load^16^. However, the spatial distribution pattern of the moisture field under constant temperature and dynamic loading is not clear. The moisture data from the test process was plotted and analyzed for its distribution pattern, as shown in Fig. 6.

At the beginning of the test, as shown in Fig. 6a, the upper soil layer had a maximum moisture content of 29.5% in the area near the ice layer and a minimum of 9.5% in the area at the soil surface, a difference of 20%. This is due to the high temperature of the upper soil during filling, which causes the surface of the ice to melt, resulting in a high moisture content. The moisture content in the bottom area of the lower soil layer is high and the moisture content in the upper layer is low. The high moisture content in the bottom of the lower soil layer is due to the sprinkling of water in the bottom of the test chamber at the beginning of the test. The low moisture content in the upper part of the upper soil layer and the upper part of the lower soil layer is due to the evaporation of water during the test.

After 48 h, as in Fig. 6b, the moisture content of both the upper and lower soil layers changed compared to 0 h. The changes were particularly pronounced in the upper soil layer, where the moisture content in the soil near the ice layer decreased and the moisture content in the area below the load increased significantly. The moisture content of the soil below the load was 19% at 0 h and increased to 25% after 48 h, an increase of 6%. The moisture content of the soil near the ice decreased from 29.5 at 0 h to 21.5% at 48 h, a decrease of 8%. The area of increased moisture content below the load is similar to the area of spatial distribution of the load stress. The moisture content of the lower soil layer increases in areas close to the ice and decreases in areas away from the ice. As the test progresses, in Fig. 6c–f, the moisture content of the soil layer changes less.

The significant change in the spatial distribution of moisture in Fig. 6b is due to the fact that the soil temperature was high at this time and the moisture in the soil did not freeze completely, and the migration of moisture in the soil occurred under the effect of temperature gradient and the dynamic load. The reason why the moisture distribution in Fig. 6c–f did not insignificant change is that as the test progressed, the moisture in the soil froze into ice, filling the pores in the soil and blocking the moisture migration channels, while on the other hand, the load caused the pores in the soil to be compressed and the porosity reduced, which could also lead to blocked moisture migration.

### Pore water pressure evolution during tests

**Figure 7:**
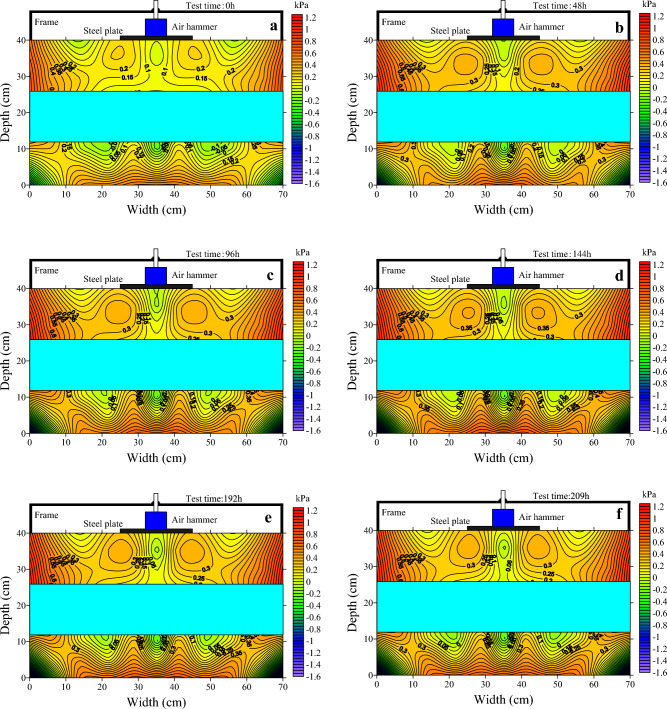
Evolution of pore water pressure over 209 h. (**a**) Pore water pressure field plot at 0 h; (**b**) pore water pressure field plot at 48 h; (**c**) pore water pressure field plot at 96 h; (**d**) pore water pressure field plot at 144 h; (**e**) pore water pressure field plot at 192 h; (**f**) pore water pressure field plot at 209 h.

It has been shown that melt soils under cyclic traffic loading gradually accumulate pore water pressure within the subgrade^17^. Under intermittent cyclic loading, the pore water pressure in the soil increases^18^. However, the pattern of pore water pressure changes in permafrost under dynamic loading is not clear. The tests were carried out to study and analyze the pore water pressure in permafrost at a constant temperature, as shown in Fig. 7.

As shown in Fig. 7a, at 0 h, the pore water pressure is 0.05 kPa in the upper soil layer under load, 0.25 kPa in the area below the sides of the load, and 0.8 kPa in the area near the wall of the test chamber. The pore water pressure in the lower soil layer was − 0.1 kPa in the areas close to the ice layer and 0.6 kPa in the bottom area. The pore water pressure distribution in the upper and lower soil layers changes after the test has been carried out for 48 h, as in Fig. 7b. The area of pore water pressure of 0.05 kPa below the load in the upper soil layer increases and an area of pore water pressure of 0 kPa occurs. The area with pore water pressure of 0.25 kPa as well as its extent also expanded. The pore water pressure distribution pattern decreases from the bottom of both sides of the soil towards the bottom of the load. The pore water pressure distribution area in the lower soil layer did not change, but the pore water pressure value increased.

During the test from 48 to 209 h, as in Fig. 7b–f, the pore water pressure area in the upper layer of soil less than 0.1 kPa gradually increased, while the area of 0.3 kPa first remained the same, then increased, then decreased and finally stabilized, but the pore water pressure pattern all decreased from the bottom of both sides of the soil towards the bottom of the load.

Throughout the test, it was found that the pore water pressure distribution in the upper soil layer changed more during 0–48 h, and changed but less during 48–209 h. The pore water pressure in the upper soil layer changed greatly during 0–48 h because the strength of the soil was small and the pore space of the soil was reduced under the action of the dynamic load, resulting in a large change in pore water pressure. As the test progresses the strength of the soil increases and the effect of the dynamic load diminishes, resulting in a reduction in the change in pore water pressure. The upper part of the lower soil layer, with the upper soil layer and the middle ice layer, is subjected to less load, so the pore water pressure changes little throughout the test.

## Discussion and analysis

From the previous analysis it is clear that for soils containing ice layers under constant temperature and dynamic loads. Dynamic loading has an effect on the temperature and moisture of the soil as well as the spatial distribution of pore water pressure. The analysis revealed that the hydrothermal as well as the pore water pressure of the soil varied considerably over a period of 48 h. In order to further understand the interaction between the hydrothermal and pore water pressures of the soil under dynamic loading. Therefore, a comprehensive analysis of the hydrothermal and pore water pressures was carried out for 0 and 48 h.

**Figure 8:**
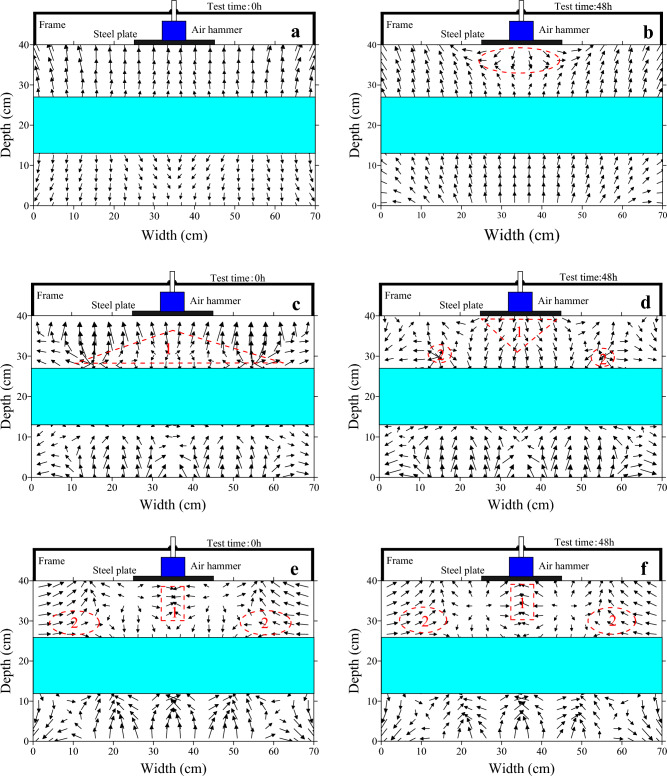
Temperature, moisture and pore water pressure vector plots. (**a**) Temperature vector plot at 0 h; (**b**) temperature vector plot at 48 h; (**c**) moisture vector plot at 0 h; (**d**) moisture vector plot at 48 h; (**e**) pore water pressure vector plot at 0 h; (**f**) pore water pressure vector plot at 48 h.

Figure 8 shows the vector plots of temperature, moisture and pore water pressure in the soil at 0 h and 48 h. In the diagram, the arrow points in the direction of the decreasing value. At 0 h in Fig. 8a, the heat in the soil is conducted from the middle to the top and bottom sides. After 48 h, the temperature in the bottom area of the specimen and the area below the load was high (red circles in Fig. 8b). The heat is conducted from the higher temperatures to the lower temperatures. In the upper soil layer, heat conduction from above downwards occurs, due to the effect of the dynamic load.

In turn, the conduction of cold has an effect on the spatial distribution of moisture in the soil, which is known to migrate from the unfrozen zone to the frozen zone (from the bottom to the top in the case of unidirectional freezing) under the influence of the temperature gradient, which in turn leads to a redistribution of moisture in the soil. However, as can be seen from the 0 h and 48 h moisture vector diagrams in Fig. 8c,d, the direction of moisture migration in the soil changes under the influence of the dynamic load. At 0 h, as in the triangular shaped area 1 in the diagram, the moisture content is large at this area, but after 48 h, the area with the large moisture content changes. At this point, area 1 below the load has a high moisture content. In addition, the moisture content of area 2, marked by the red dashed line in the diagram, is also large, but decreases relative to the corresponding area at 0 h. The migration of moisture in the soil from area 2 to area 1 resulted in an increase in the moisture content of area 1. Comparing the pore water pressure vector diagram at 0 h (Fig. 8e) and 48 h (Fig. 8f), it was found that during the period 0-48 h, the pore water pressure in the area corresponding to the moisture vector diagram b) was high in area 2 and low in area 1, and there was a pressure difference between the pore water pressure in area 2 and area 1. Under the effect of the pore water pressure difference, the moisture in the soil migrated from area 2 to area 1, resulting in an increase in moisture in area 1 and a decrease in moisture in area 2.

**Figure 9:**
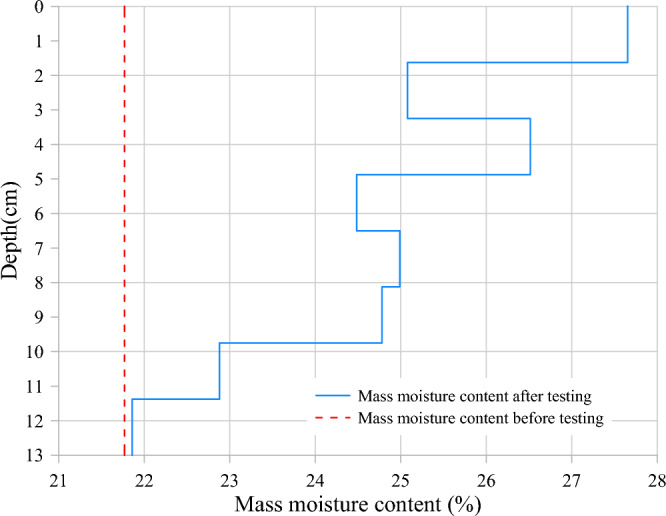
Change in mass moisture content in soil with depth.

To verify the change in moisture content of the soil under load, samples were taken from area 1 after test and the mass moisture content of the samples was determined using the drying method. It is clear from Fig. 9 that the moisture content of the soil below the load increased after the test compared to before the test and gradually decreased with increasing depth, which corresponds to Fig. 8d as well as Fig. 6. The analysis of the temperature, moisture and pore water pressure vector soils in the soil revealed that the application of dynamic loads changed the direction of migration of moisture in the soil, which in turn affected the spatial distribution of moisture in the soil. The reason for this is that the application of dynamic loads changes the spatial distribution of pore water pressure in the soil, resulting in pressure differences between different zones, which affect the migration of moisture.

The constant temperature and dynamic load test revealed that the temperature, moisture and pore water pressure all changed significantly at the beginning of the test under dynamic load. As the temperature of the soil layer decreased, the moisture and pore water pressure changed less, indicating that the effect of dynamic loading was weakened. Therefore, the temperature of the soil base should be controlled for road foundations built in areas with ice layers, and when the soil temperature is low, the effect of dynamic loading can be reduced or weakened.

In addition, as the loading time in the test was 0.5 s and the load loading interval was 1 s, further research is required to determine whether changing the loading time and the loading interval will have an effect on the results. Research is also needed on the hydrothermal state in multi-layered ice- layer soils under dynamic loading.

## Conclusion

The hydrothermal processes in the ice-layers soil were investigated by constant temperature and dynamic loading. It was found that the application of dynamic loads altered the hydrothermal and pore water pressure states in the soil. The application of dynamic loads leads to a pore water pressure difference between different areas of the soil sample, where the migration of moisture is altered by the pressure difference, eventually leading to the formation of areas with higher moisture content in the area below the load. However, the reduction in soil temperature will weaken the effect of the load, therefore, the temperature of the soil should be controlled for frozen subgrade with ice layer to prevent the accumulation of moisture in the soil.

## Data Availability

Some or all data generated or used during the study are available from the corresponding author by request.

## References

[1] Zhang H, Zhang Z, Zhang K, Xiao D, Zhang L (2019). Effects of freeze-thaw on the water-heat process in a loess subgrade over a cut-fill transition zone by laboratory investigation. Cold Reg. Sci. Technol..

[2] Wu XY, Niu FJ, Lin ZJ, Luo J, Zheng H (2018). Delamination frost heave in embankment of high speed railway in high altitude and seasonal frozen region. Cold Reg. Sci. Technol..

[3] Niu F, Zheng H, Li A (2020). The study of frost heave mechanism of high-speed railway foundation by field-monitored data and indoor verification experiment. Acta Geotech..

[4] Wang SJ, Chen ZG, Qin WJ, Yu LM (2013). Mechanism analysis of subgrade frost heaving in seasonally frozen regions. J. Hway. Transp. Res. Dev. (English Edition)..

[5] Lin Z, Niu F, Li X, Li A, Liu M (2018). Characteristics and controlling factors of frost heave in high-speed railway subgrade, Northwest China. Cold Reg. Sci. Technol..

[6] Dou MJ (2000). Discussion on permafrost stability along the Qinghai-Tibet Highway. J. Glaciol. Geocryol. (in Chinese)..

[7] Xiao AN, Yue Q, Jin-Tao XU (2015). Numerical research on low embankment settlement subjected to freeze-thaw cycle and dynamic vehicle loading actions. Highway..

[8] Cheng PF, Yin CY (2014). Research on the classification of frost swelling of road base clay in seasonal freezing area. J. China Foreign Highway..

[9] Wang TH (2005). Analysis of frost heave deformation on subgrade in permafrost regions. China J. Highways Transp..

[10] Iwata, & Shingo. Driving force for water migration in frozen clayey soil. *Soil Sci. Plant Nutr*. **26**(2), 215–227 (1980).

[11] Guodong C (1983). The mechanism of repeated-segregation for the formation of thick layered ground ice. Cold Reg. Sci. Technol..

[12] Xu, X., & Deng, Y. *Experimental study of moisture migration in permafrost*. 29, 36–94 (Beijing: Science press, 1991).

[13] Wen, D.Y., Jiang, N.S., Zhang, W.Y., Li, H., Li, Y.X. Dynamic stress-strain response law of frozen soil in cold regions under temperature rise and dynamic Load. *Saf. Environ. Eng*. **28**(4), 29–34 (2021).

[14] Shen ZY, Zhang JY (1997). Dynamic strength characteristics and failure criterion of frozen silt. J. Glaciol. Geocryol. (in Chinese)..

[15] Zhang, C.Q., He, P., Roman, *et al.* Long-term strength evaluation under vibration load [C]. In *Annual report of State Key Laboratory of Frozen Soil Engineering: 1993. Lanzhou: Lanzhou Institute of Glaciology and Geocryology*, Chinese Academy of Sciences. 165–172 (1993).

[16] Xiao D, Wei MA, Zhao S, Zhang Z, Feng W (2017). Research on changing laws of pore water pressure and moisture field in soil subjected to the combination of freeze-thaw and static load actions. J. Hunan Univ. (Natural Sciences)..

[17] Wang L, Zhang L, Wang T, Zhang S (2023). Investigation of water and soil migration and mud pumping of subgrades under traffic load. Atmosphere..

[18] Cheng, C. C. Research on dynamic characteristics of Hangzhou soft clay under intermittent cyclic loading on macro and micro scales. (Doctoral dissertation, Zhejiang University) (2019).

